# Immune Complex Signatures of Patients with Active and Inactive SLE Revealed by Multiplex Protein Binding Analysis on Antigen Microarrays

**DOI:** 10.1371/journal.pone.0044824

**Published:** 2012-09-11

**Authors:** Krisztián Papp, Péter Végh, Renáta Hóbor, Zoltán Szittner, Zoltán Vokó, János Podani, László Czirják, József Prechl

**Affiliations:** 1 Department of Immunology, Eötvös Loránd University, Budapest, Hungary; 2 Diagnosticum Ltd., Budapest, Hungary; 3 Department of Rheumatology and Immunology, Clinic Center, University of Pécs, Pécs, Hungary; 4 Department of Health Policy and Health Economics, Eötvös Loránd University, Budapest, Hungary; 5 Syreon Research Institute, Budapest, Hungary; 6 Department of Plant Systematics, Ecology and Theoretical Biology, Eötvös Loránd University, Budapest, Hungary; 7 Immunology Research Group of the Hungarian Academy of Sciences, Eötvös Loránd University, Budapest, Hungary; Weizmann Institute of Science, Israel

## Abstract

Systemic lupus erythematosus is characterized by dysfunctional clearance of apoptotic debris and the development of pathogenic autoantibodies. While the complement system is also involved in the disease no attempt has been made to generate a comprehensive view of immune complex formation from various autoantigens. We increased the complexity of autoantibody profiles by measuring the binding of two complement proteins, C3 and C4, in addition to two antibody classes, IgG and IgM, to a collection of autoantigens. These complement components covalently bind to those microarray features where antibodies and other serum components induce complement activation. Using this technology, we compared functional serum antibody profiles of control subjects (n = 31) and patients with lupus erythematosus (n = 61) in the active (n = 22) and inactive (n = 39) phase of the disease. Multivariate analysis was applied to identify contributions of binding data on 25 antigens to the discrimination of the study groups. Receiver operating characteristic analysis was used to portray the discriminative property of each measured parameter for each antigen in pairwise group comparisons. Complement C3 and C4 deposition increased on autoantibody targets in spite of the decreased serum complement concentrations, and decreased on other autoantigens, demonstrating the imbalance of complement function in patients with lupus erythematosus. Our observations confirmed previously known markers of disease and showed that C3 and C4 deposition data were at least as powerful as Ig binding data in separating the study groups.

## Introduction

Systemic lupus erythematosus (SLE) is a pleomorphic autoimmune disease characterized by the formation of immune complexes, which trigger inflammation and lead to tissue destruction, multi-system organ dysfunction and premature mortality. Establishing the diagnosis of SLE requires the fulfillment of several defined criteria [1], involving multiple laboratory measurements. The presence of antinuclear antibodies (ANA) is a hallmark of lupus, which along with additional serological tests is used to establish diagnosis; of the latter dsDNA specific IgG is highly specific for SLE [2]. Patients, especially those in clinical remission, may however lack these autoantibodies. Thus, even though several SLE biomarkers are in use, there is still need for novel ones that would improve the diagnosis and monitoring of the disease [3].

The complement system is involved both in the development of SLE and in mediating pathological effects of autoantibodies [4]. While the lack of early components predisposes to disease, immune complex initiated complement activation promotes inflammation and leads to secondary deficiency of complement components [5]. These net alterations in serum complement are also used for following the disease course [6]. SLE-associated in vivo complement activation can also be monitored by measuring soluble split products [7] and cell-bound products [8], [9], [10]. Direct correlation between disease activity and the ex vivo ability of pathological antibodies to fix complement has been suggested by several authors [11], [12], [13], an exception being [14].

We have recently shown that complement fixation can be easily monitored in antigen specific fashion using antigen microarrays [15] and that the technology is suitable to track complement activating properties of anti-nuclear antibodies in mice [16]. In this paper we describe complement C3 and C4 deposition patterns in control non-autoimmune subjects and lupus patients in the active and inactive phase of the disease and demonstrate the utility of such measurements.

## Results

### Characteristics of Patients with SLE

The presence of antinuclear antibodies and also of anti-phospholipid antibodies was confirmed in the SLE groups (Table 1). Decreased total C4 and C3 concentrations in the SLE groups indicated the consumption of complement components. Serum C4 concentrations in patients with active disease were significantly lower than in patients in the inactive phase of the disease.

**Table 1:** Characteristics of the study groups.

Attribute	Control	Active SLE	Inactive SLE
male:female ratio (n)	1∶30 (31)	1∶21 (22)	4∶35 (39)
Age (years)	47 (37–54)	41 (31–53)	49 (37–58)
Disease duration (years)	0	5 (3–13)	6 (2–15)
Kidney damage (%)	0%	38%	0%
ANA (U/ml)	1.5 (1.4–2.1)	27.6 (16.5–275.0)^a^	15.5 (3.2–254.4)^a^
Anti-dsDNA (U/ml)	5.15 (3.9–6.5)	12.9 (4.9–41)^a^	8.2 (5.6–24.3)^a^
Anti-Sm (U/ml)	1.2 (1.0–1.6)	1.7 (1.3–2.6)^a^	1.3 (1.1–2.0)
Anti-β2GP (U/ml)	1.1 (0.9–1.6)	1.9 (1.1–5.5)^a^	1.8 (1.4–2.6)^a^
Serum C3 (g/l)	1.4 (1.1–1.7)	1.0 (0.8–1.2)^a^	1.1 (1.0–1.3)^a^
Serum C4 (g/l)	0.25 (0.19–0.34)	0.14 (0.08–0.17)a,b	0.17 (0.12–0.22)a,b

Medians and interquartile ranges are shown, where applicable. Significant differences in serological measurements from the control group.

a and between the two SLE groups.

b are indicated (p<0.05, Mann-Whitney U test). β2GP, β2-glycoprotein.

### Identification of Immune Complex Components that Best Separate the Tested Populations

**Figure 1:**
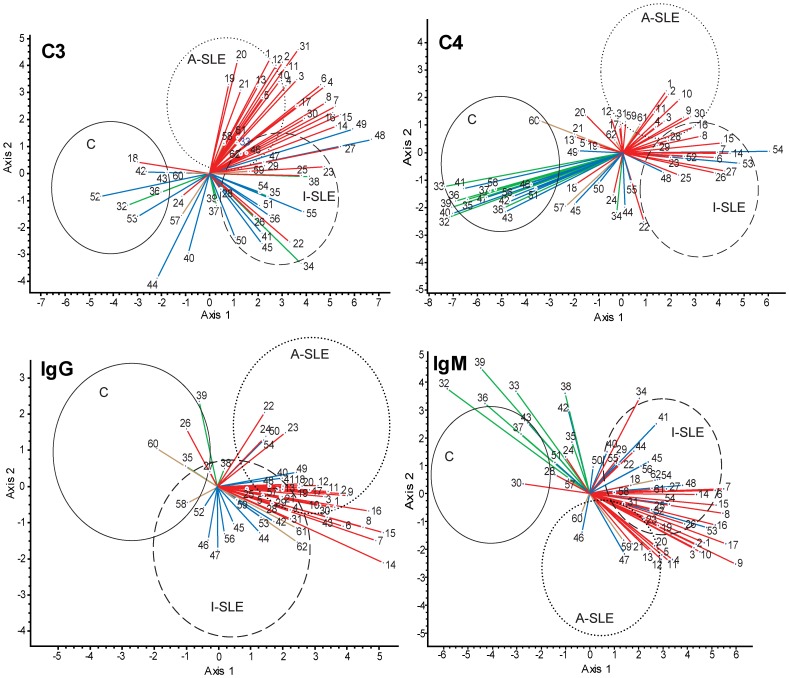
Canonical variates analysis (CVA) of binding data. Binding data of C3, C4, IgG and IgM were used to generate a canonical space, defined by axis 1 and axis 2, from the discriminant functions derived through eigenanalysis. Coordinates of observations for the three study groups (control, C; active SLE, A-SLE; inactive SLE, I-SLE) in this canonical space are enclosed within the ellipsoids with a 95% confidence. Vectors represent correlations of variables (antigens) with the canonical axes and are superimposed on the ordination of observations: the length and directionality of these vectors offer a possibility to evaluate the relative influence of antigens upon the separation of groups. For example, the vector of C3 binding data of antigen 8 separates group A-SLE and I-SLE from group C but not A-SLE and I-SLE from each other. Antigen groups are color-coded: all nuclear materials (nucleic acids and nuclear proteins), red; collagens, green; complement, blue; lipids, brown. The complete list of antigens shown here is found in Table 2.

The binding of four different immune complex components, C3, C4, IgG and IgM was determined for each antigen on the microarray, following incubation with the tested sample. Thus, four sets of binding data were generated, corresponding to these four serum proteins with immunological function. To identify the contribution of the various antigen binding events of each of these four proteins to the separation of control subjects and patients with active and inactive SLE, we used the multivariate method of canonical variates analysis (CVA). Autoantigens with known association to SLE and complement proteins (shown in Table 2) were included in the analysis with the aim of supplementing and comparing known antibody binding phenomena with complement deposition data. Since the number of canonical axes is one less than the number of groups, in our case CVA produced scores in two dimensions. Ellipsoids in Figure 1 enclose the regions where 95% of the observations of the indicated groups are located provided that sampling was random and the distribution of variables is normal (see Figure S1 for the distribution of observations). The two SLE groups and the controls are best separated in all four cases by the nuclear antigens. The collagen antigens are negatively correlated with nuclear antigens in the case of C4 and IgM, but this contrast diminishes for C3 and IgG binding measurements. Lipids and complement proteins are not responsible for any separation as a set of antigens. From this evaluation of antigen sets, it is obvious that classical IgG type antibodies were present in most of the patients, as confirmed by ANA testing. While IgM antibodies with nuclear specificity characterize both SLE groups, collagen specific IgM was identified as marker of healthy subjects, mainly present in the control group. A similar pattern was observed for C4: its deposition on nuclear components is increased in the SLE groups, while C4 deposition on collagens is stronger in the control group. C3 data resemble IgG binding data in the sense that deposition on nuclear antigens increased in patients with SLE. For comparison, data derived from routine clinical laboratory tests were also subjected to CVA, those 14 variables could not separate the three study groups (Figure S2).

**Table 2:** List of antigens used for CVA.

Antigen set	Number	Antigen name
Nucleic acids (red)	1–5	dsDNA
	6–9	ssDNA
	10–13	plasmid DNA
	14–17	RNA
	18–21	chromatin
Nuclear proteins (red)	22–23	histone II-A
	24–25	histone III-SS
	26–27	Jo-1 antigen
	28–29	La (SSB)
	30–31	Ro (SSA)
Collagens (green)	32–33	collagen pI
	34–35	collagen pIX
	36–37	collagen pVI
	38–39	collagen sI
Complement (blue)	40–41	C1q
	42–43	C3
	44–45	factor H
	46–47	factor I
	48–49	factor P
	50–51	factor B
	52–54	C4
	55–56	vitronectin
Lipids (brown)	57–58	phosphatidyl-serine
	59–60	phosphatidyl-ethanolamine
	61–62	cardiolipin

Antigens were used in two or more dilutions to study different epitope densities, higher numbers of the same antigen indicate dilution steps. Color coding in Figure 1 is indicated in parentheses.

**Figure 2:**
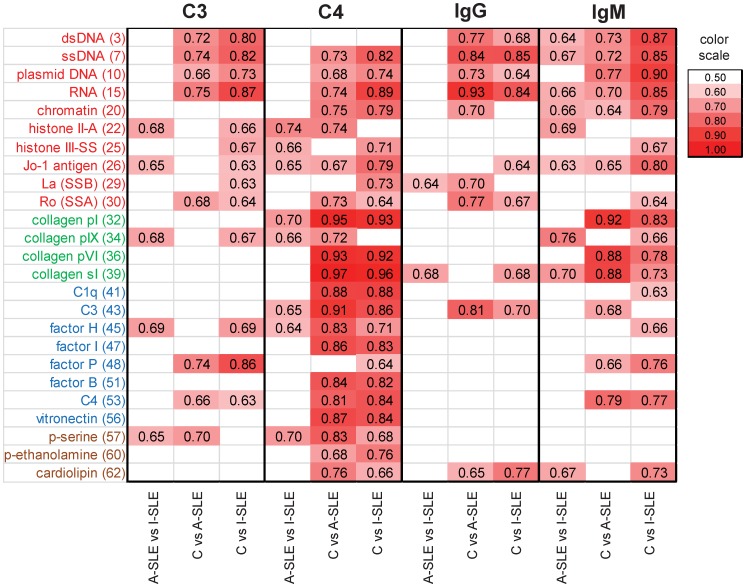
Discriminative properties of antibody binding and complement deposition. The three study groups (control, C; active SLE, A-SLE; inactive SLE, I-SLE) were compared in every pair using ROC analysis for each of the four measured proteins. AreaUnder the Curve (AUC) values for antigen binding events that were found to have significant (p<0.05) discriminative properties are shown in the form of a heat map. Antigen dilution points with the highest summarized AUC values are shown.

As Figure 1 suggests, some measurements appear to contribute strongly to the separation of the SLE groups from the control group. It is more difficult to find antigen binding events that differentiate the inactive phase from the active phase of the disease (see Figure S3,S4 for paired CVA comparisons). In addition to multivariate analysis, we also performed receiver operator characteristic (ROC) analysis, to characterize the discriminative power of each measurement for pairs of groups (Fig. 2). The pattern of discriminative properties of C4 most closely resembles that of IgM, while C3 is comparable to IgG. The figure also shows that fewer interactions differentiate the active and inactive form of the disease and even these interactions have poorer discriminative properties.

### Nucleic Acids

#### C3 deposition on nucleic acids is characteristic of SLE

**Figure 3:**
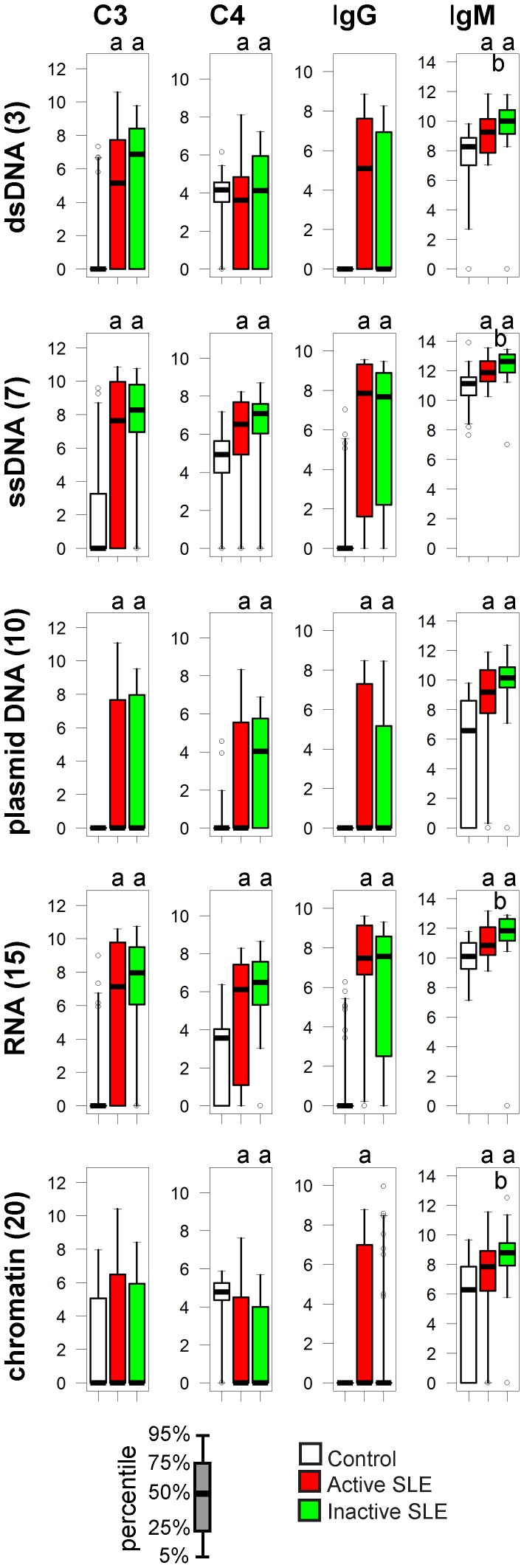
Alterations of immune complex composition on nucleic acid antigens. A. Bar charts depict the reactivity profile of the three study groups, four components of immune complexes shown separately. Scale of y axis is relative fluorescence units. ^a^p<0.05 compared to control group, ^b^p<0.05 comparing active to inactive SLE Empty circles represent outliers: samples exceeding the upper or lower quartile values with 1.5-times the interquartile distance.

Microarray results confirmed the presence of anti-dsDNA IgG, along with reactivity against ssDNA, plasmid DNA, RNA and chromatin. C3 deposition on purified nucleic acids was minimal or absent in the control group but appeared in both inactive and active SLE patients (Fig. 3), resembling the specificity of pathological IgG. C3 deposition was observed on chromatin even in the control group, with only a trend of elevation in the active disease group, in spite of the increased levels of anti-chromatin IgG.

#### C4 deposition increases or decreases in an antigen-dependent manner

Early complement components, preceding the C3 cleavage stage, are normally found bound to nucleic acids and promote swift removal of cellular debris. Accordingly, a baseline C4 deposition was measurable in all three groups. In spite of the decreased overall C4 concentrations (Table 1) the amount of relative deposited C4 was increased on ssDNA, plasmid DNA and RNA or remained unchanged on dsDNA in SLE patients (Fig. 3). Immune complexes formed on chromatin contained decreased amounts of C4 in the disease groups.

### Nuclear Protein Antigens

In addition to nucleic acids, other nuclear molecules are also targets of autoantibodies in SLE. While usually found in the cytoplasm and mainly a target in myositis, we also include here histidyl-tRNA synthetase or Jo-1 antigen, because it is present in the nucleus as well and SLE patients have been reported to be positive for this autoantibody.

**Figure 4:**
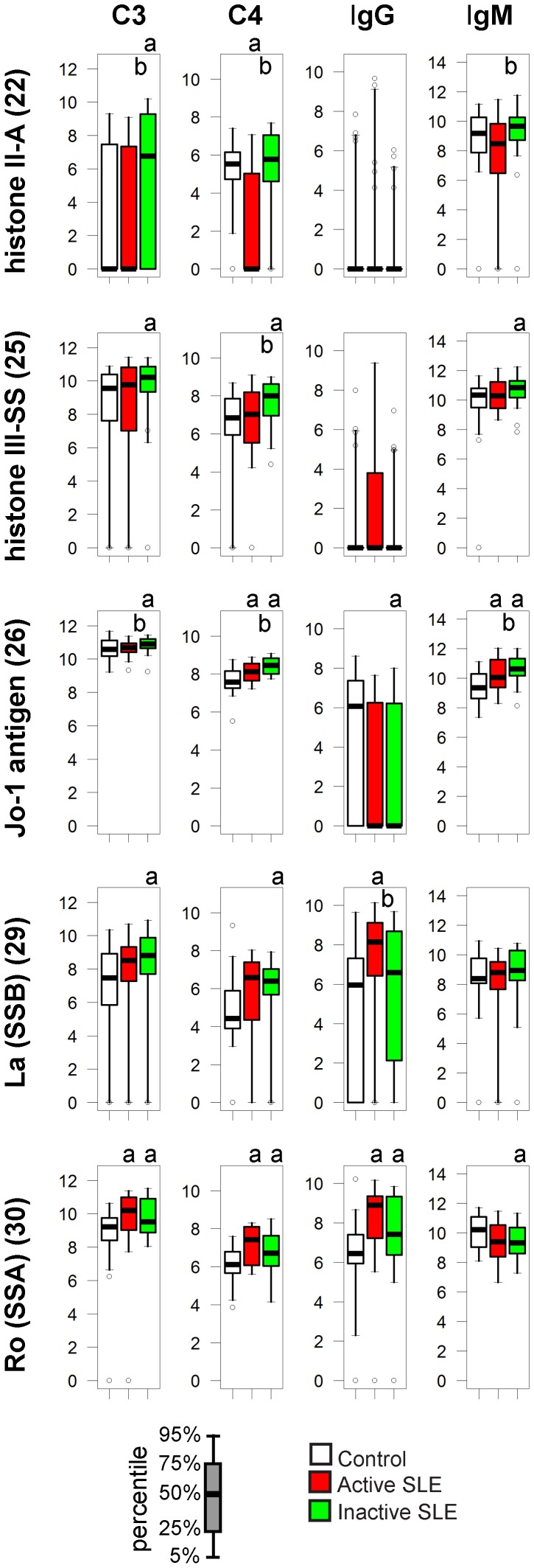
Alterations of immune complex composition on nuclear protein antigens. See Figure 3. for details.

Anti-histone II-A and III-SS IgG antibodies were observed in some cases, most of them in active SLE patients, but were not representative for any group (Fig. 4). Increased C3 deposition on histone II-A and III-SS was characteristic of inactive SLE patients. Modest but significant elevations in C3, C4 and IgM binding to Jo-1 characterize SLE patients while IgG binding ranges overlap in all three study groups (Fig. 4). IgG but not IgM binding to SSA and SSB was observed in SLE patients with the highest values being observed in patients with active disease and concomitant increase in C3 and C4 deposition.

### Lipids

**Figure 5:**
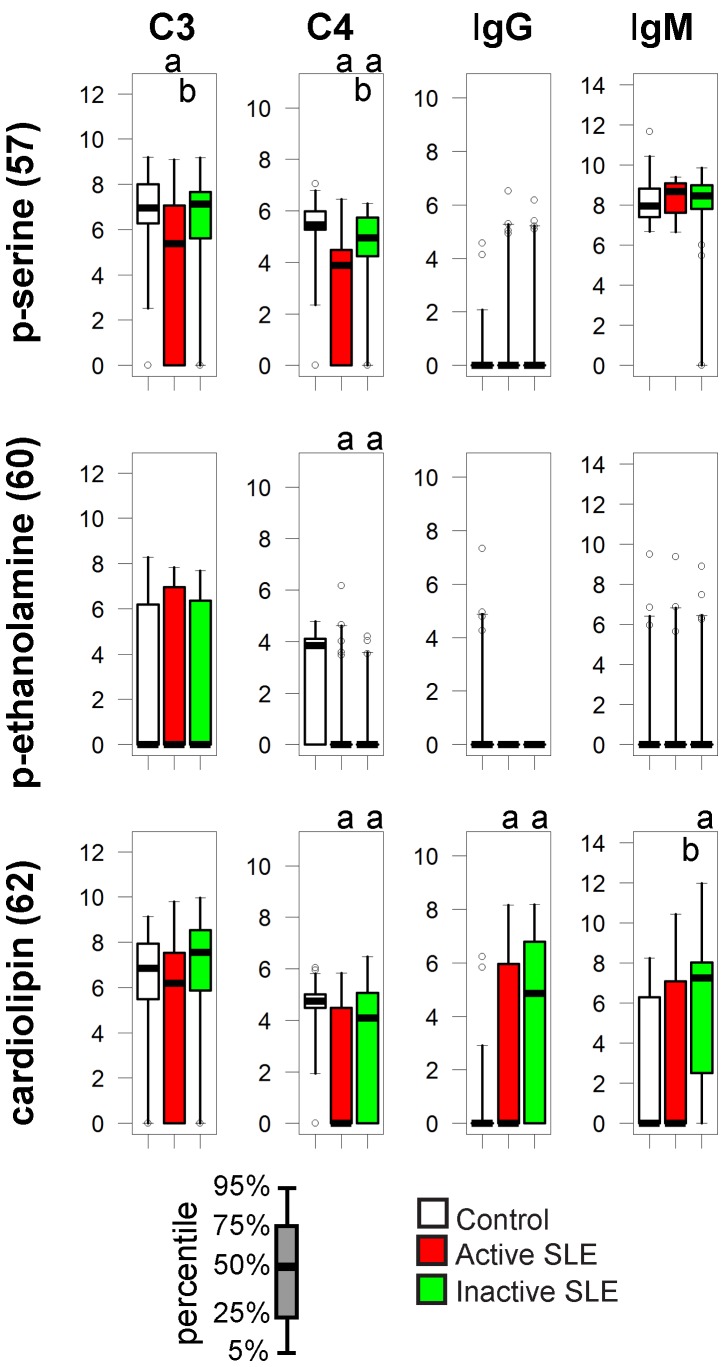
Alterations of immune complex composition on cardiolipin. See Figure 3. for details.

Antibodies against phospholipids and beta-2-glycoprotein can develop in SLE patients and may manifest as antiphospholipid syndrome. In our assay significantly increased levels of IgG and IgM autoantibodies against cardiolipin were detected in the inactive SLE group, with reduced C4 deposition on all three lipids in both SLE groups (Fig. 5).

### Collagens

**Figure 6:**
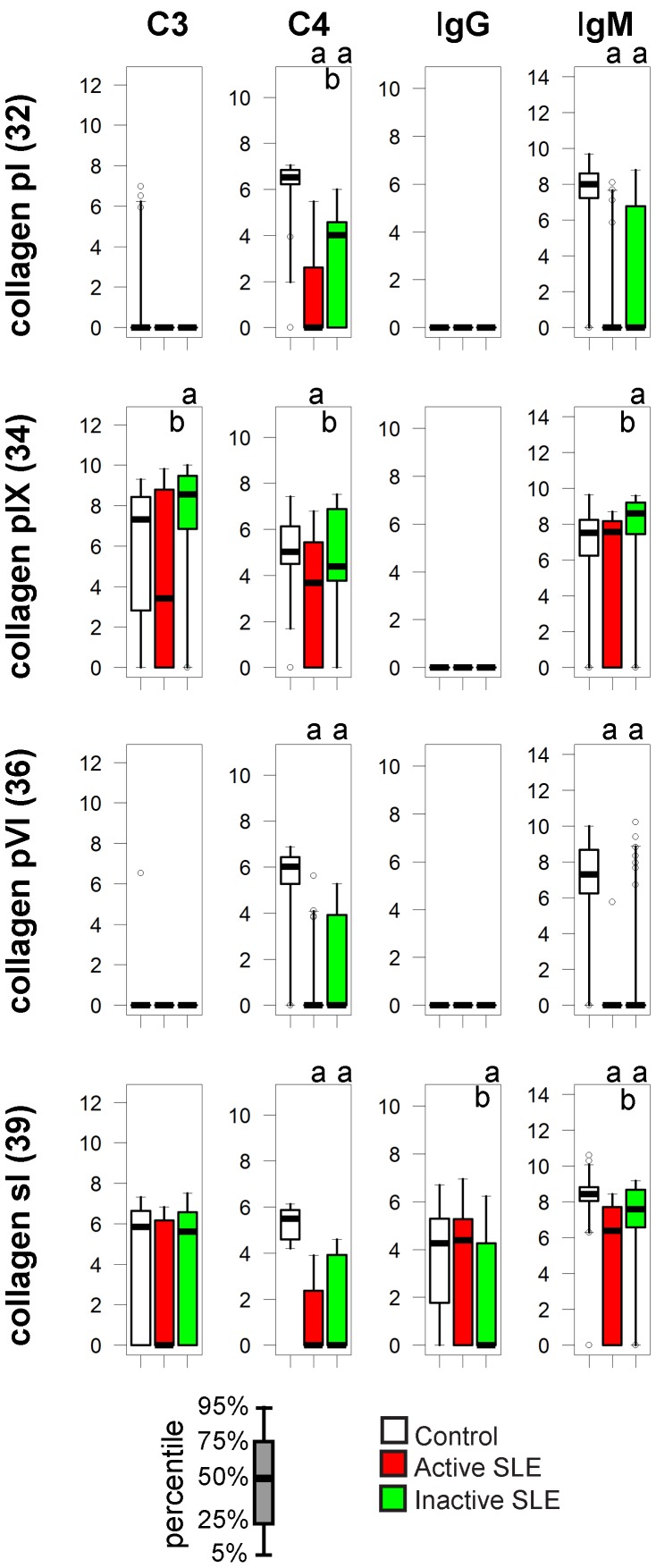
Alterations of immune complex composition on collagens. See Figure 3. for details.

The classical technical term “connective tissue disease” for the group of diseases that include SLE refers to the fact that the extracellular matrix is involved in the pathogenesis of these conditions. Even though anti-collagen IgG has been observed in SLE, our measurements could not confirm increased IgG binding. Instead, it was the decrease in IgM binding and in C4 deposition that characterized especially patients with active SLE (Fig. 6). Interestingly C3 binding to collagen pIX was increased rather than decreased in the inactive SLE group.

### Complement Components

**Figure 7:**
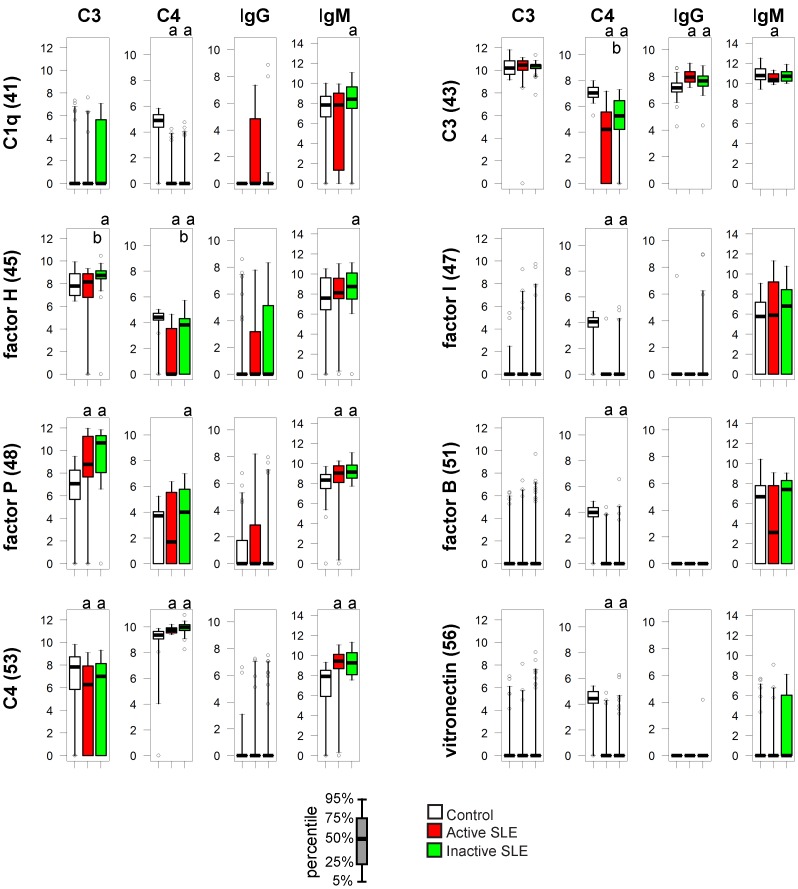
Alterations of IC composition on complement proteins. See Figure 3. For details.

Complement function is strongly affected in SLE: levels of early complement components show primary or secondary decrease and autoantibodies against various components or complexes (such as the nephritic factor against C3bBb) have been described. We observed major alterations in the composition of protein complexes formed on C1q when treated with sera from SLE patients (Fig. 7). These complexes contained significant amounts of IgM and moderate amounts of C4 in control samples. In the SLE sera C4 signals were mostly below the detection level; bound IgM showed significant increase in the inactive SLE group and a tendency to decrease in patients with active disease. Additionally, anti-C1q IgG positive samples were found predominantly in patients with active disease, though this was not a significant alteration for the group.

Factor P or properdin was included as a control protein that stabilizes the C3bBb complex and therefore promotes alternative pathway activation. Modest levels of C4 binding to properdin were also observed unexpectedly, in all three study groups. Pronounced increase in C3 binding and increased IgM binding was measured in both SLE groups. Decreased C4 binding to other complement proteins, such as factor H, factor I, factor B, vitronectin and C3 is consistent with decreased total serum C4 concentrations in the SLE groups. It is also due to the decreased C4 activity in SLE groups that normalization results in increased C4 signals on printed C4 spots.

## Discussion

Current diagnostic assays for autoimmune diseases monitor global changes in complement activity that accompany the appearance of autoantibodies. In contrast to this global view, the measurement of complement interactions on antigen microarrays provides a much more detailed view of the humoral immune system, which resolves complement consumption to the level of antigens. Such a technology could be ideally implemented for SLE diagnosis, because of the involvement of a multitude of autoantigens and of complement in the disease. Protein microarray approaches already identified autoantigen clusters associated with SLE and autoantibody profiles that could improve diagnostic accuracy [17], [18], [19]. No attempt has been made, however, to understand antibody-induced effector functions, such as complement activation, in a multiplex fashion.

In our assay, the binding patterns of both immunoglobulins (IgG, IgM) and complement proteins (C4, C3) were capable of separating the control non-autoimmune group from the disease groups and also the two disease groups (Fig. 1,2). In fact, the separation between the control and the SLE groups is the weakest for IgG, more emphasized for IgM and C3, and the sharpest for C4 (Fig. 1). Thus, complement deposition data from antigen microarrays carry relevant information and could be used to improve the discriminative power of IgG determinations. Discriminant functions derived from further, larger training data sets could be used to determine the group membership of a new patient, based on his/her microarray scores, the success of this assignment depending on how sharply the *a priori* defined groups differ in the canonical space.

Earlier studies assessed complement fixing ability of anti-DNA antibodies in correlation with nuclear fluorescence pattern [11], development of lupus nephritis [12], [14], [20], [21], and subclass ratios [22]. Although not unanimously, most results suggested that complement fixing ability of anti-DNA antibodies was strongly related to anti-DNA titers and often to active kidney disease. These observations are in line with the theory that high affinity pathological anti-dsDNA antibodies cause inflammation and tissue destruction at least partly via complement activation. Our results only partly support such a scenario: deposition of C3 on dsDNA is observed almost exclusively in SLE patients (Fig. 3) yet this deposition was not further increased in patients showing signs of disease activity. On the contrary, C3 deposition measurement on nucleic acids identified inactive SLE patients with higher confidence than active SLE patients (Table S1, Fig. 2). dsDNA specific IgG titer correlates with disease activity [19], implying that its sensitivity for identifying patients in the inactive phase of the disease should be weaker. Combination of anti-dsDNA measurement with dsDNA C3 deposition should therefore identify more patients. Indeed, combining these to measurements consistently increased AUC values for all three nucleic acids, and confidence level in the case of dsDNA, in discriminating inactive patients from control subjects (Table S1).

Quantitative analysis of C3 fixation by DNA-antibody immune complexes suggests that C3b binds IgG and not the DNA molecule itself [23]. IgG binding to dsDNA can therefore not only trigger complement C3 cleavage but also serve as an acceptor molecule. Elevated levels of anti-nuclear IgM in inactive SLE patients (Fig. 3) can initiate C3 cleavage and may result in C3 binding to pathological IgG that is also present in the complex, perhaps at levels that are otherwise not detectable. Another possibility is that C3 deposition is more pronounced in inactive patients since global complement activity is less affected than in patients with active disease. This situation is not supported by an animal model of SLE where C3 deposition only developed with the progression of disease activity [16].

The role of autoreactive IgM in disease pathogenesis is elusive. DNA specific IgM has been suggested to have protective effect [24] or precede the development of overt disease [18]. Our studies show that IgM specific for all kinds of antigens containing nucleic acids is increased in SLE subjects, indicating that it is not protective but rather a marker of disease. A possible reason for the discrepancy between our observations and of others’ is the methodological difference, since the lower dilution of serum what we use is beneficial for the detection of lower affinity IgM binding.

Anti-phospholipid antibodies of IgG and IgM class can appear in lupus patients. The pattern of cardiolipin reactivity (Fig. 5) is an example of increased binding of Igs with decreased C4 deposition on the antigen. The net outcome is the relative increase of proinflammatory stimuli for cells via FcγRs.

Decreased binding of IgM to collagen has been reported by others [17] who also used moderately diluted serum with the intent of capturing natural antibodies. There are two possible reasons behind this observation: impaired synthesis of natural antibodies binding to collagen or the increased consumption of these antibodies. B-cell development is abnormal in SLE patients on the one hand, while tissue inflammation can expose extracellular matrix and could lead to the exhaustion of the natural IgM on the other hand, leaving the question open for further investigations.

C1q specific IgG was only observed among SLE subjects and mostly in the active group, reflecting that this reactivity is a marker of global SLE activity [25]. Decrease in C4 deposition reflects impaired classical pathway activity in the SLE subjects. We included factor P, an initiator and stabilizer of alternative pathway activity [26], in the panel of reference proteins of the array to monitor alternative pathway activity in the serum samples. Increased IgM binding along with stronger C3 deposition on properdin in the SLE group was an unexpected finding. Alternative pathway activation is known to contribute to tissue injury in lupus and other diseases [27]; our observation confirms an imbalance between the alternative and classical pathways in SLE.

In summary, determination of complement deposition can be carried out simultaneously with the measurement of immunoglobulin binding to autoantigens. In addition to revealing the complexity of pathological immune complex formation accompanying disease, such tests could be easily introduced into the diagnostic algorithms used today. How measurement of complement deposition could help in resolving diagnostic dilemma of lupus versus other diseases needs further investigations.

## Materials and Methods

### Study Subjects

The study was approved by the national Scientific and Research Ethics Board (reference number 25563-0/2010-1018EKU); written informed consent was obtained from each participant. Serum samples were stored at −70°C until use. Control serum samples of subjects without known autoimmune conditions were selected from the repository of the Drug Research Center and were matched in gender and age. SLE patients fulfilled the international criteria [1]. Based on the disease activity, patients were divided into active and inactive subgroups. As an active sign of disease we took any of the following clinical symptoms: polyarthritis, inflammatory skin symptoms, serositis, clinical signs of active central nervous system and kidney manifestation. Beside at least one of the above mentioned symptoms, clinically active patients also had increased erythrocyte sedimentation rate and/or fever. In addition to microarray measurements, all samples were further characterized by routine laboratory tests (Table 1).

### Microarray Production and Measurements

58 different antigens (see list in GEO database) were spotted onto nitrocellulose-covered FAST slides (GE Healthcare) using BioOdyssey Calligrapher miniarrayer (BioRad). Different dilutions of the antigens were printed in triplicates then stored at 4°C in sealed bags. Dried arrays were rinsed in PBS for 15 minutes before use, and then incubated with diluted serum at 37°C for 1 hour, providing suitable conditions for complement activation. For each patient 5-fold diluted serum was used in two different arrays: one for the detection of bound C3 and IgM, the other for IgG and C4. Sera were diluted in 5% BSA, 0.05% Tween 20, Ca^2+^- and Mg^2+^-supplemented Veronal buffer. Serum treated slides were washed with PBS containing 0.05% Tween 20, then incubated in the mixture of 1∶5,000 diluted Alexa647-conjugated F(ab’)2 fragment goat anti-human C3 antibody (Cappel) and 1∶5,000 diluted Cy3-conjugated F(ab’)2 fragment goat anti-human IgM (mu chain speific) (Jackson ImmunoResearch) or 1∶2,500 diluted FITC-conjugated goat anti-human C4 (Cappel) and 1∶2,500 diluted APC-conjugated F(ab’)2 fragment goat anti-human IgG (gamma chain specific) (Jackson ImmunoResearch). Labeling with fluorescent antibodies was carried out at room temperature for 30 minutes in PBS containing 5% BSA and 0.05% Tween 20. After washing in PBS containing 0.05% Tween 20, arrays were dried and scanned by Typhoon Trio+ imager (Amersham Bioscience).

### Analysis of Microarray Data

After visual inspection data were analyzed with Genepix software. Signal intensities were calculated by subtracting background from medians of three parallel signal intensities in a spreadsheet program (Microsoft Excel). Signals not exceeding two standard deviations of local background signals on a slide were clamped to an arbitrary value of 1. For interassay comparison, data were normalized to the average of two dilutions of selected control materials among all slides. Protein G, anti-human IgM, human IgG and protein G features on arrays were used for normalization of C3, IgM, IgG and C4 signals, respectively. These reference points are expected to be saturated with similar kinetics in all samples and were therefore rendered all equal post-normalization, shifting the entire dataset of a slide accordingly. This adjustment compensated for both overall biological variations in the samples and technical differences in detection. The data discussed in this publication have been deposited in the National Center for Biotechnology Information’s Gene Expression Omnibus (GEO) [28] and are accessible through GEO series accession number: GSE26768 (www.ncbi.nlm.nih.gov/geo/query/acc.cgi?acc).

### Statistical Analysis

Laboratory test results were compared using Mann-Whitney U test. Differences were considered statistically significant when p<0.05, as indicated in the figures and tables.

A multivariate procedure, CVA (alias discriminant function analysis) was used for the interpretation of microarray-derived data. CVA maximizes separation of *a priori* defined groups of observations and is useful when variables best discriminating between the groups of patients are to be identified. A partial limitation of CVA is that the number of variables (antigens) cannot exceed the number of observations (patients). Therefore, a subset of antigens was selected on a logical basis: those that were known to be associated with SLE. The results of CVA are canonical scores obtained from the discriminant functions derived through eigenanalysis, which serve as coordinates of observations in the canonical space. Correlations of variables (antigens) with the canonical axes are illustrated by vectors superimposed on the ordination of observations: the length and directionality of these vectors offer a possibility to evaluate the relative influence of antigens upon the separation of groups. CVA was carried out for all four binding datasets (C3, C4, IgG, IgM) separately. Computations were performed by the SYN-TAX 2000 program package [29].

Afterwards the univariate method of ROC was applied to the variables (GraphPad Prism, GraphPad Software Inc. La Jolla, CA), to determine the efficiency of discrimination between the study groups. Significance is reported when AUC was different from the expected 0.5 value with a confidence interval of 95%.

## Supporting Information

Figure S1 **CVA with sample coordinates.** Results from microarray measurements are shown with the positions of the individual samples in the three groups of control (C, red), active lupus (A-SLE, black) and inactive lupus (I-SLE, blue) subjects. Unlike in Figure 1, antigens are not color coded and are without number codes here, for the sake of clarity.
(TIF)

Figure S2 **CVA of routine laboratory tests.** Results from clinical laboratory ELISA and nephelometry tests were analyzed just the way microarray results were. The figure shows that these test cannot separate the three groups of control (C), active lupus (A-SLE) and inactive lupus (I-SLE) subjects.
(TIF)

Figure S3 **CVA using two groups, with active and inactive SLE merged.** Correlation coefficients of the four types (C3, C4, IgM, IgG) of antigen interaction data and the canonical variates are shown, with sample scores on the variates below. Antigen numbering and coloring is as in Table 2.
(TIF)

Figure S4 **CVA comparing active verus inactive SLE.** Correlation coefficients of the four types (C3, C4, IgM, IgG) of antigen interaction data and the canonical variates are shown, with sample scores on the variates below. Antigen numbering and coloring is as in Table 2.
(TIF)

Table S1 **Comparison of sample classification strategies.** Samples were grouped into ANA negative and positive subsets, then further divided based on the indicated measurements (first two columns). Cut-off values providing 100% specificity were chosen for all measurements, thus all positives are true positives for SLE in the table. Discriminative properties of the different measurements with respect to the indicated groups were also compared by ROC analysis (*p<0.05, **p<0.01, ***p<0.001). ^a^ dsDNA IgG was only tested in 20 control subject but is expected negative in the whole group. n.a., not applicable; NA, all three nucleic acids.
(PDF)
