# Supplementary figures and images for: Immune Complex Signatures of Patients with Active and Inactive SLE Revealed by Multiplex Protein Binding Analysis on Antigen Microarrays
Source: PLoS One. 2012 Sep 11;7(9):e44824. doi: 10.1371/journal.pone.0044824 (PMC3439431; doi:10.1371/journal.pone.0044824)

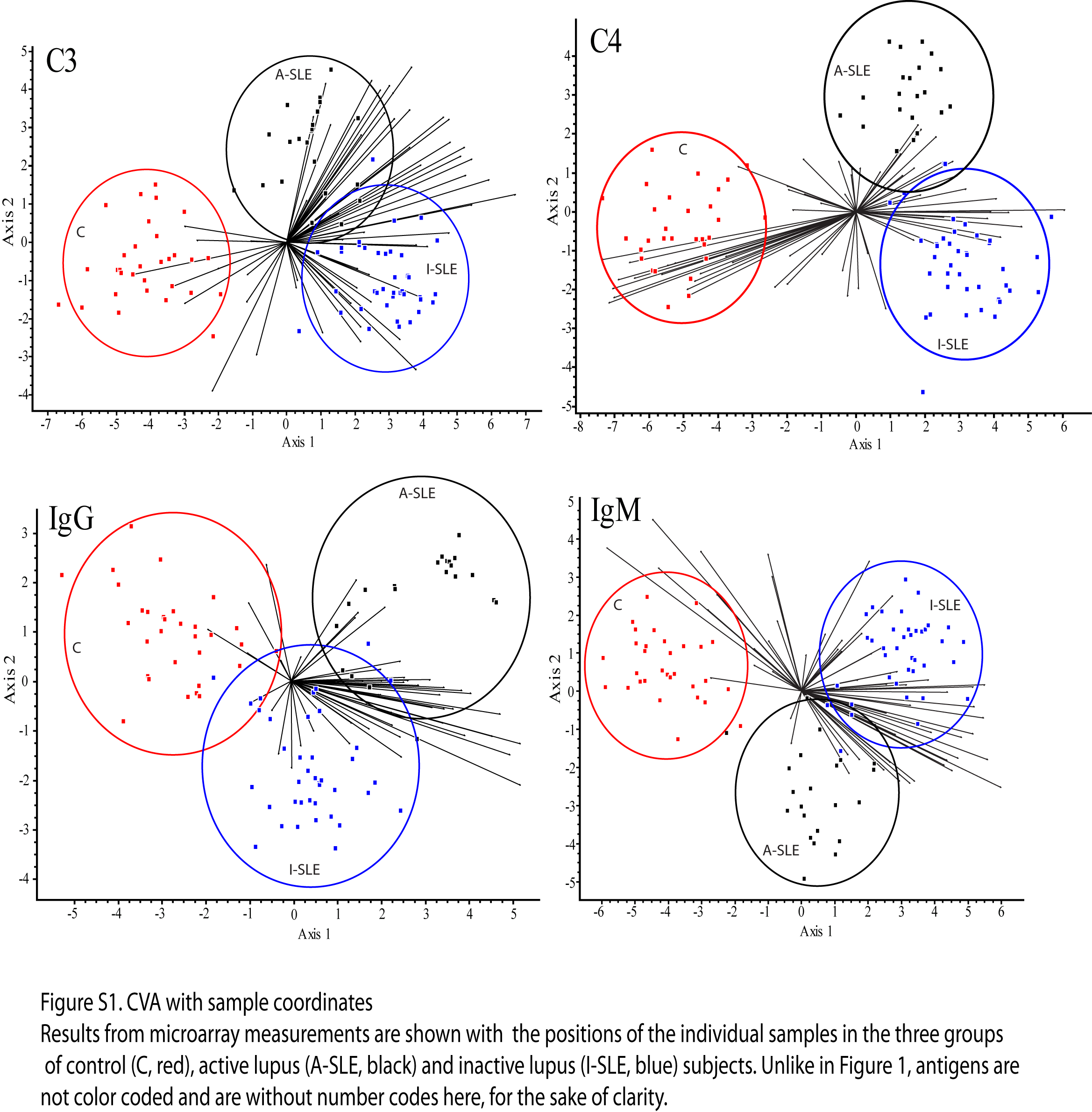

Supplement: Figure S1 — CVA with sample coordinates. Results from microarray measurements are shown with the positions of the individual samples in the three groups of control (C, red), active lupus (A-SLE, black) and inactive lupus (I-SLE, blue) subjects. Unlike in Figure 1, antigens are not color coded and are without number codes here, for the sake of clarity. (TIF) [file pone.0044824.s001.tif]

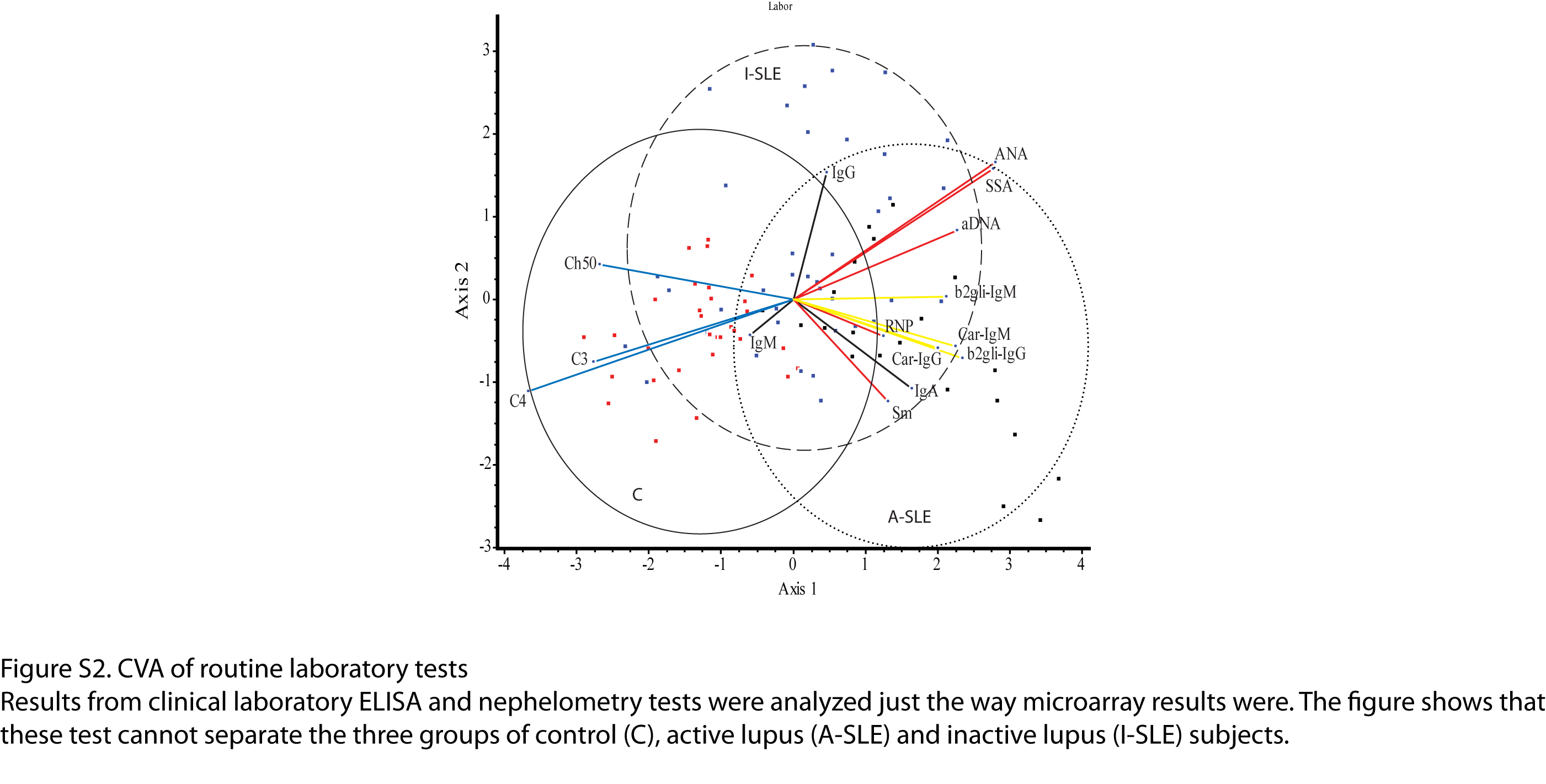

Supplement: Figure S2 — CVA of routine laboratory tests. Results from clinical laboratory ELISA and nephelometry tests were analyzed just the way microarray results were. The figure shows that these test cannot separate the three groups of control (C), active lupus (A-SLE) and inactive lupus (I-SLE) subjects. (TIF) [file pone.0044824.s002.tif]

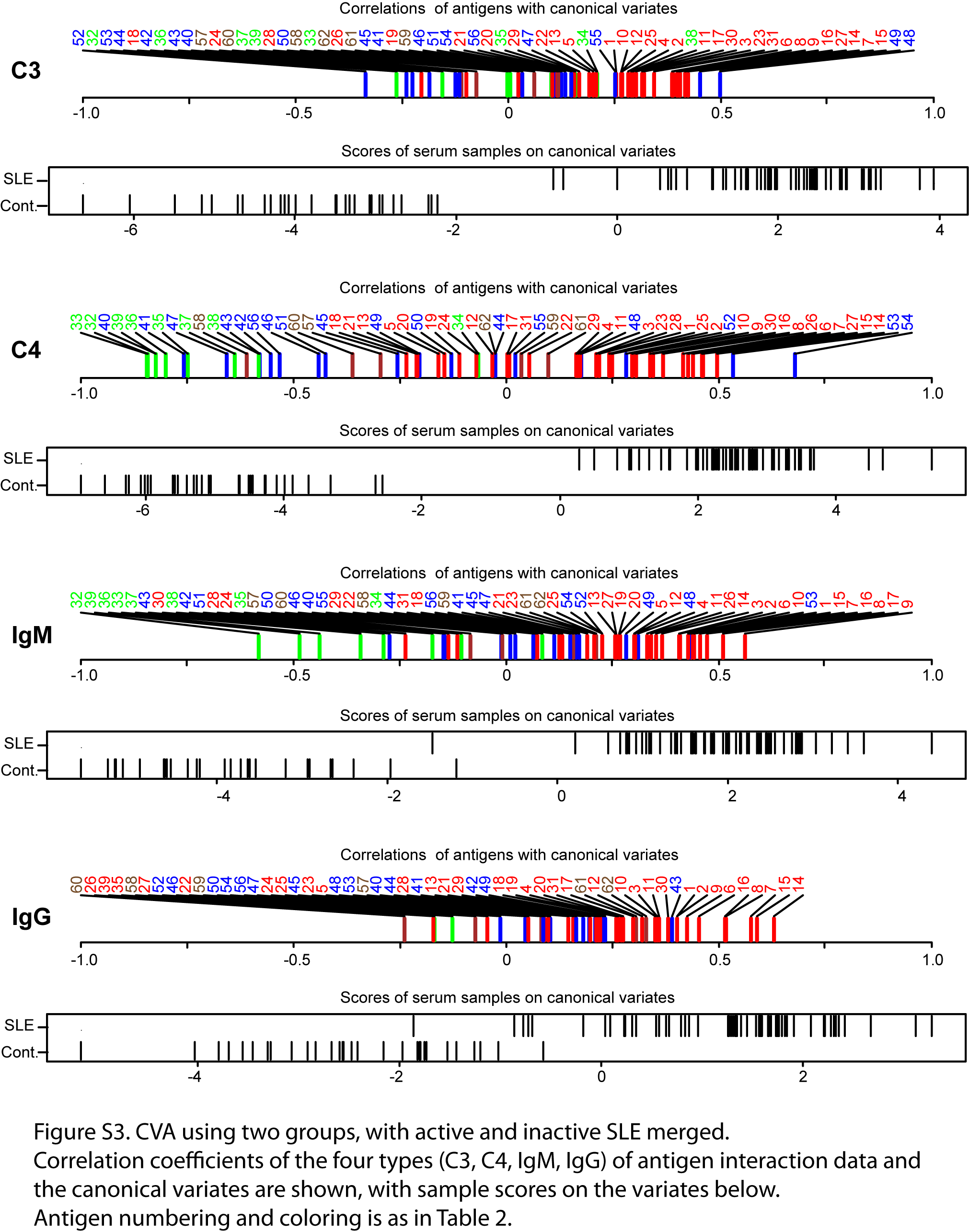

Supplement: Figure S3 — CVA using two groups, with active and inactive SLE merged. Correlation coefficients of the four types (C3, C4, IgM, IgG) of antigen interaction data and the canonical variates are shown, with sample scores on the variates below. Antigen numbering and coloring is as in Table 2. (TIF) [file pone.0044824.s003.tif]

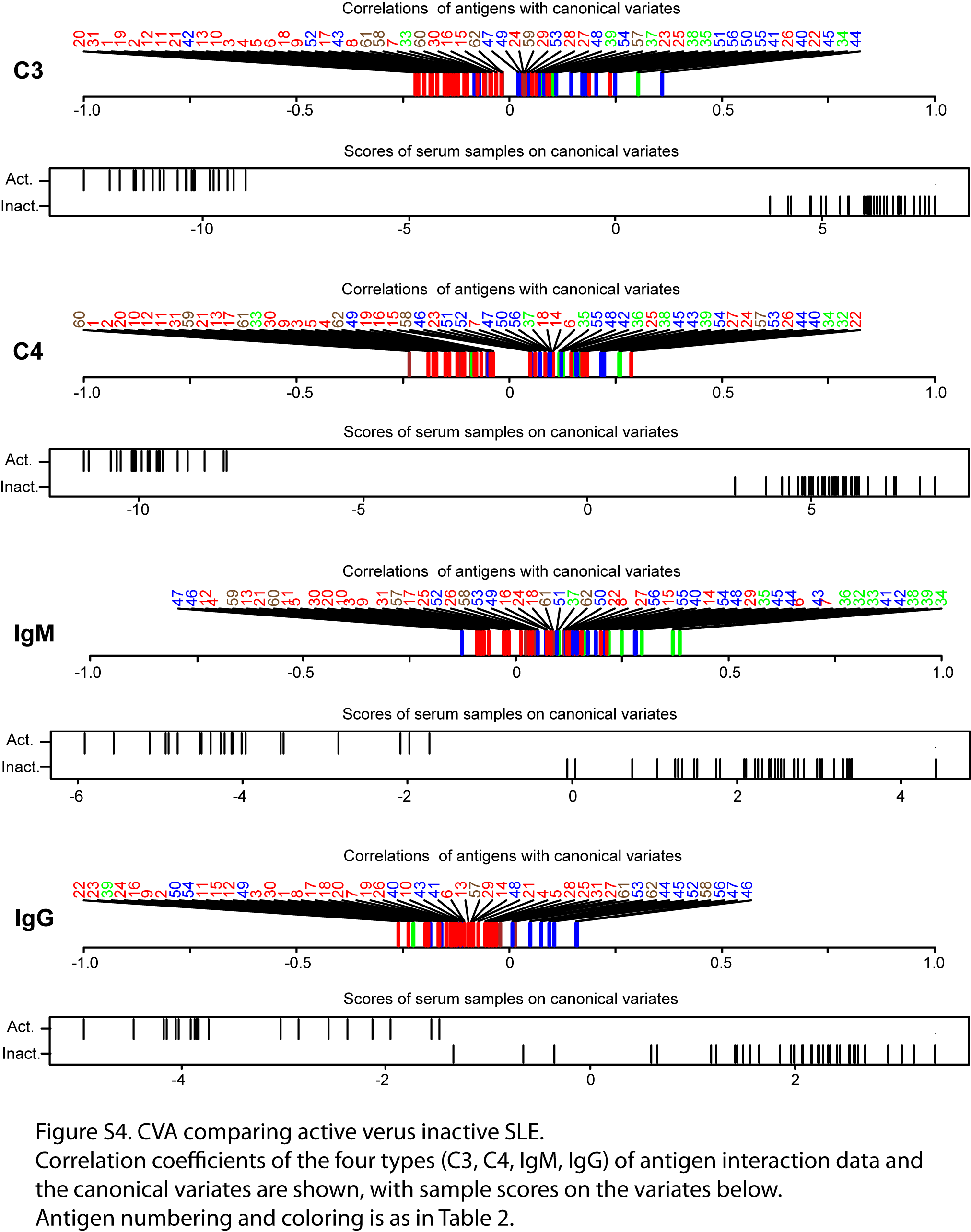

Supplement: Figure S4 — CVA comparing active verus inactive SLE. Correlation coefficients of the four types (C3, C4, IgM, IgG) of antigen interaction data and the canonical variates are shown, with sample scores on the variates below. Antigen numbering and coloring is as in Table 2. (TIF) [file pone.0044824.s004.tif]
