# Supplementary material for: Immune Complex Signatures of Patients with Active and Inactive SLE Revealed by Multiplex Protein Binding Analysis on Antigen Microarrays
Source: PLoS One. 2012 Sep 11;7(9):e44824. doi: 10.1371/journal.pone.0044824 (PMC3439431; doi:10.1371/journal.pone.0044824)
Supplement: Table S1 — Comparison of sample classification strategies. Samples were grouped into ANA negative and positive subsets, then further divided based on the indicated measurements (first two columns). Cut-off values providing 100% specificity were chosen for all measurements, thus all positives are true positives for SLE in the table. Discriminative properties of the different measurements with respect to the indicated groups were also compared by ROC analysis (*p<0.05, **p<0.01, ***p<0.001). a dsDNA IgG was only tested in 20 control subject but is expected negative in the whole group. n.a., not applicable; NA, all three nucleic acids. (PDF) [file pone.0044824.s005.pdf]

**Table S1**. Comparison of sample classification strategies.

| Test method | positive : negative ratios | | ROC AUC | |
|---|---|---|---|---|
| | ANA+ | ANA- | C : A | C : I |
| *clinical status* | *51:0* | *9:29* | *n.a.* | *n.a.* |
| dsDNA ELISA | 30:21 | 1:20(37)[a] | 0.71[*] | 0.73[**] |
| dsDNA-IgG | 26:25 | 0:38 | 0.77[***] | 0.68[*] |
| dsDNA-C3 | 23:28 | 0:38 | 0.75[**] | 0.79[***] |
| dsDNA-IgG&C3 | 31:20 | 0:38 | 0.81[***] | 0.81[***] |
| ssDNA-IgG | 34:17 | 7:31 | 0.85[***] | 0.84[***] |
| ssDNA-C3 | 15:36 | 2:36 | 0.75[**] | 0.81[***] |
| ssDNA-IgG&C3 | 35:16 | 7:31 | 0.87[***] | 0.89[***] |
| RNA-IgG | 10:41 | 2:36 | 0.90[***] | 0.82[***] |
| RNA-C3 | 21:30 | 3:35 | 0.74[**] | 0.87[***] |
| RNA-IgG&C3 | 25:26 | 4:34 | 0.89[***] | 0.91[***] |
| NA-IgG | 44:7 | 7:31 | 0.89[***] | 0.83[***] |
| NA-C3 | 34:17 | 3:35 | 0.75[**] | 0.88[***] |
| NA-IgG&C3 | 46:5 | 7:31 | 0.88[***] | 0.91[***] |

Samples were grouped into ANA negative and positive subsets, then further divided based on the indicated measurements (first two columns). Cut-off values providing 100% specificity were chosen for all measurements, thus all positives are true positives for SLE in the table. Discriminative properties of the different measurements with respect to the indicated groups were also compared by ROC analysis ([*]p<0.05, [**]p<0.01, [***]p<0.001).
[a] dsDNA IgG was only tested in 20 control subject but is expected negative in the whole group. n.a., not applicable; NA, all three nucleic acids
